# Characterization of transmitted drug resistance among recently infected HIV-1 men who have sex with men in Hebei Province, China

**DOI:** 10.1038/s41598-025-26028-7

**Published:** 2025-11-11

**Authors:** Xinli Lu, Shuofan Dong, Lin Ma, Ning An, Guangyi Bai, Meng Liu, Xueang Xu, Yingying Wang, Yan Li, Qi Li

**Affiliations:** Department of AIDS research, Hebei Key Laboratory of Pathogen and Epidemiology of Infectious Disease, Hebei Provincial Center for Disease Control and Prevention, No. 97 Huai’an East Road, Yuhua Distinct, Shijiazhuang, 050021 Hebei China

**Keywords:** HIV-1, Recent infections, TDR, MSM, Diseases, Medical research, Microbiology

## Abstract

Despite transmitted drug resistance (TDR) surveillance is the critical role in HIV-1 prevention strategies, epidemiological data remain scarce for newly infected men who have sex with men (MSM) in Hebei Province, China. To address this data gap, we conducted a cross-sectional study involving 173 MSM recently infected with HIV-1. Plasma samples were subjected to HIV-1 RNA extraction, followed by amplification and sequencing of the *pol* gene region (PR-RT: 1.3 kb; IN: 0.8 kb). In this study, 173 sequences were successfully sequenced, and the sequencing success rate was 92.5% (173/187). Subtyping analysis revealed a predominance of CRF07_BC (36.4%, 63/173) and CRF01_AE (32.3%, 56/173), with a notable proportion of unique recombinant form (URF, 20.2%, 35/173). The overall TDR prevalence was 7.51% (13/173), categorized by drug class as follows: protease inhibitors (PIs, 2.31%, 4/173), nucleoside reverse transcriptase inhibitors (NRTIs, 1.16%, 2/173), non-nucleoside reverse transcriptase inhibitors (NNRTIs, 2.31%, 4/173), and integrase strand transfer inhibitors (INSTIs, 2.89%, 5/173). Notably, one participant exhibited concurrent resistance to NRTIs, NNRTIs, and INSTIs. While these findings suggest moderate TDR control in Hebei’s MSM population, we emphasize the necessity for sustained surveillance to dynamically assess treatment program efficacy, optimize second-line therapy regime, and guide public health interventions. This study provides the first comprehensive TDR profile for recently infected individuals in Hebei, offering critical insights for regional HIV-1 management.

## Introduction

Globally, the number of recent HIV infections amounted to 1.3 million in 2023. Although this was a 39% decrease from the 2010 figure of 2.1 million, there is still a long way to go to reach the goal of reducing the number of recent infections to 370,000 by 2025^1^. The number of recent HIV infections among MSM (men who have sex with men) increased by 11% per year between 2010 and 2022. It is estimated that 210,000 recent HIV infections occurred in 2022 alone^2^.

Compared with previous studies on the prevalence of recently diagnosed HIV infections, the caculation of the prevalence rate ‌using recently HIV-infected individuals‌ (defined as those who contracted HIV within the past 130 days) as the study population ‌avoids‌ potential time lag effects, ‌reflects‌ the current epidemic status, and ‌enhances‌ the accuracy of forecasting HIV epidemic trend.

Since the ‌National Free Antiretroviral Treatment Program (NFATP)‌ was fully implemented in China in 2004^3^, the treated people have progressively expanded. In 2016, China aligned with World Health Organization (WHO)’s‌ “Treat All” strategy^4^, extending ‌ antiretroviral therapy (ART)‌ coverage to all diagnosed HIV individuals. Although expanded ART has reduced HIV-related morbidity and mortality, prolonged treatment durations have given rise to the increase of drug resistance rates. ‌HIV drug resistance‌ elevates treatment costs and complexity, substantially increases ART failure risks^5^, accelerates the transmission of resistant strains, and critically challenges AIDS containment efforts.

WHO classifies HIV drug resistance into three categories: transmitted drug resistance (TDR), pretreatment drug resistance (PDR), and acquired drug resistance (ADR)^6^. TDR refers to resistance detected in ART - naïve individuals infected with drug - resistant strains. In China, the national HIV control framework mandates that all of recently diagnosed cases are registered in the Comprehensive AIDS Control Data Information Management System (CACDIMS). The initiation of ART is required within 10 working days after diagnosis, with all treatment records systematically archived in the national AIDS database. Given that recently diagnosed HIV individuals have no prior exposure to ART drugs, this surveillance mechanism ensures that the detected resistance in this population is presumptively categorized as TDR, providing a critical foundation for monitoring primary transmission dynamics of resistant variants.

In resource - limited situation, the systematic monitoring of HIV ‌TDR, including baseline surveillance and resistance testing, in China remains inadequately implement compared to standardized protocols in the U.S. and Europe^7^. MSM are disproportionately affected by drug resistance‌ due to complex behavioral and biological factors^8^, whcih can severely constrain the efficacy of first - line drugs. To evaluate subtype distribution and TDR prevalence among ‌recently HIV infected MSM‌ in Hebei Province, China, we performed a population - based analysis using 2023 surveillance data from the provincial AIDS registry. This study aims to inform targeted prevention strategies and optimize ART guidelines for high - risk groups in resource - limited regions.

## Results

### Demographic characteristics

A total of 187 recently infected HIV-1 MSM were included in this study. The age distribution showed a predominance of 25–49 years (48.66%, 91/187), followed by ≤ 24 years (33.69%, 63/187) and ≥ 50 years (17.65%, 33/187). Educational attainment exhibited a bimodal distribution: 36.36% (68/187) had an educational level of junior high school or lower, and 41.18% (77/187) had a college degree or higher. Demographic characteristics revealed that the majority was Han Chinese (93.58%, 175/187), 53.48% (100/187) were unmarried, and 35.29% (66/187) were classified as workers/farmers. 22.99% (43/187) had CD4 + T - cell counts ≥ 500 cells/mm³ without obvious immunodeficiency, while 69.52% (130/187) demonstrated viral loads > 10,000 copies/mL (Table 1).

**Table 1:** Basic information of 187 recent HIV-1 infections in the MSM population in Hebei Province, 2023.

Variable	Cases	Constituent Ratio (%)
Age (years)		
≤24	63	33.69
25–49	91	48.66
≥50	33	17.65
Ethnicity		
Han	175	93.58
Others	12	6.42
Marital Status		
Unmarried	100	53.48
Married or with a spouse	61	32.62
Divorced or widowed	26	13.90
Educational Attainment		
Junior high school and below	68	36.36
High school or secondary technical school	42	22.46
College and above	77	41.18
Occupation		
Student	29	15.51
Worker/Farmer	66	35.29
Service staff/Clerk	30	16.04
Housework and unemployed	45	24.06
Others	17	9.10
CD4 Count (cells/mm³)		
200 < CD4 ≤ 349	88	47.06
350 ≤ CD4 ≤ 499	56	29.95
CD4 ≥ 500	43	22.99
Viral Load (copies/mL)		
1000 ≤ VL ≤ 5000	40	21.39
5001 ≤ VL ≤ 10,000	17	9.09
10,001 ≤ VL ≤ 100,000	86	45.99
VL>100,000	44	23.53

### Genotyping‌

**Fig. 1:**
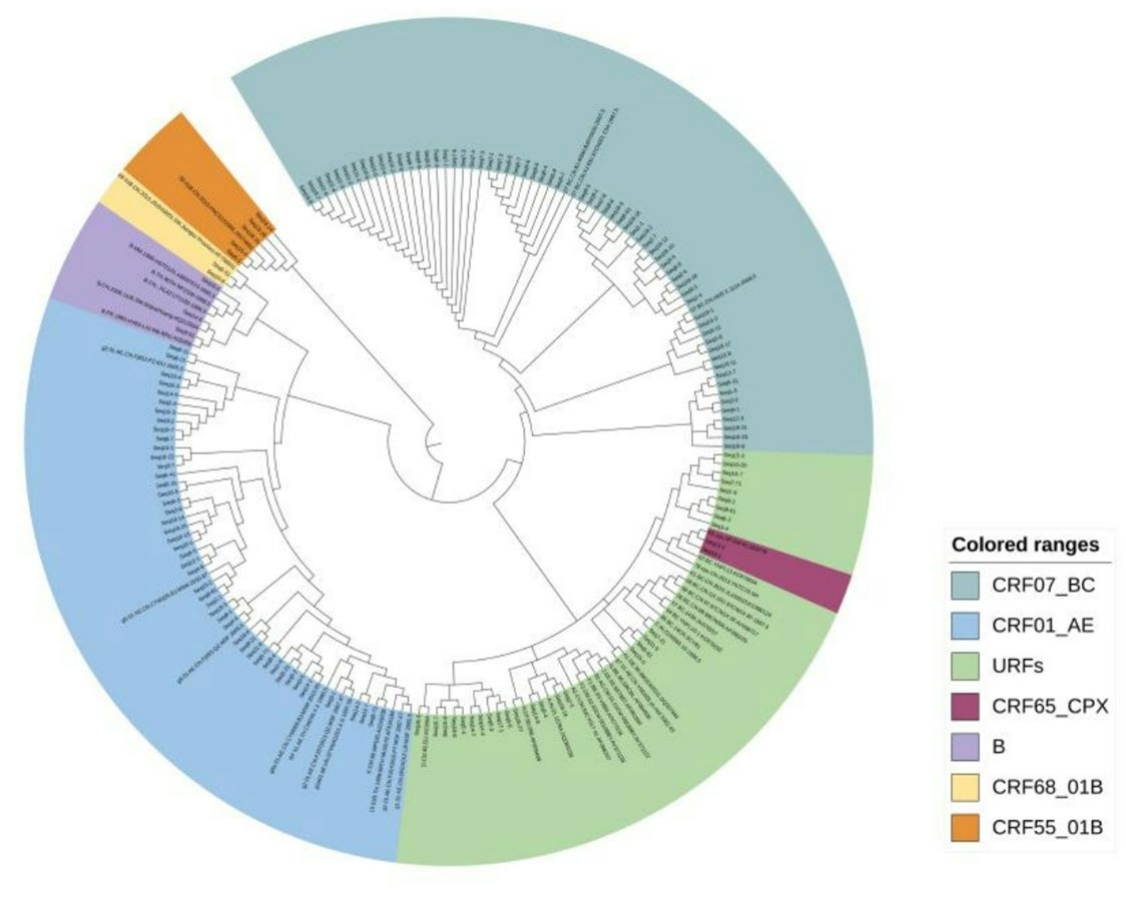
Phylogenetic tree analysis based on HIV-1 *pol* gene sequences obtained from this study. The neighboring-joining tree was constructed using MEGA 6.0 with 1000 bootstrap replicates. The reference sequences (A–D, F–H, J, K, O, CRF01_AE) were obtained from the HIV database (http://www.hiv.lanl.gov/content/index).

**Fig. 2:**
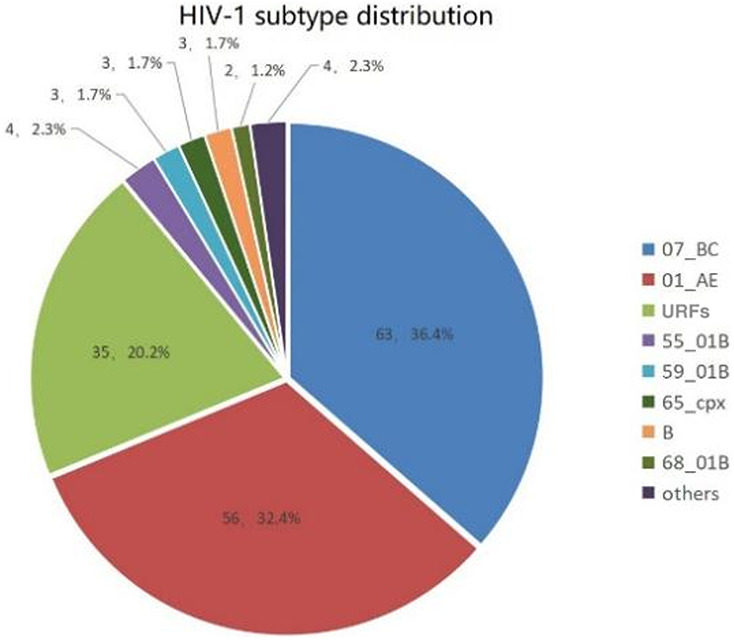
Subtype distribution of recent infected HIV-1 among MSM in Hebei province.

In this study, the gene coding regions of protease/reverse transcriptase (1.3 kb) and integrase (0.8 kb) in the *pol* region from 173 recently infected individuals were successfully amplified. The sequencing success rate was 92.5% (173/187). Figure 1 indicated that 12 kinds of subtypes were found in this study in total. Of them, the predominant subtypes identified were CRF07_BC (36.4%, 63/173) and CRF01_AE (32.3%, 56/173). URFs accounted for 20.2% (35/173), representing a significant proportion. Additionally, the following subtypes were also detected among recently infected MSM in Hebei Province: CRF55_01B (2.3%, 4/173), B (1.7%, 3/173), CRF59_01B (1.7%, 3/173), CRF65_cpx (1.7%, 3/173), CRF68_01B (1.1%, 2/173), CRF53_01B (0.5%, 1/173), CRF54_01B (0.5%, 1/173), CRF79_0107 (0.5%, 1/173), and CRF120_0107 (0.5%, 1/173) (Fig. 2).

The chi-square test showed that there were significant differences in the distribution of HIV-1 subtypes among MSM with different characteristics: the distribution of CRF01_AE showed obvious difference in different occupational populations; subtype B differed among different CD4 + T-cell count strata; and the distribution of URFs exhibited significant differences in different marital status groups (Table 2).

The chi-square trend test was conducted again. It was found that the distribution of CRF01_AE in different occupational groups showed significant difference (χ² = 17.84, *P* < 0.001). The distribution of URFs in different marital statuses indecated obvious different (χ² = 6.37, *P* = 0.040). And,, the distribution of subtype B in different CD4 counts groups was different (χ² = 4.76, *P* = 0.039), and participants with 350–499 cells/mm³ had the highest proportion of subtype B.

**Table 2:** Distribution of HIV-1 subtypes in different groups of recent infected individuals.

Sample Characteristics		Number of Cases (n, %)									
		01_AE	07_BC	55_01B	59_01B	65_cpx	68_01B	B	others	URFs	Total
Age, years	≤ 24	22(36.7)	23(38.3)	1(1.7)	1(1.7)	0	1(1.7)	1(1.7)	2(3.3)	9(15.0)	60.00
	25–49	26(30.2)	30(34.9)	3(3.5)	2(2.3)	1(1.2)	0	2(2.3)	2(2.3)	19(22.1)	86.00
	≥ 50	8(29.6)	10(37.0)	0	0	2(7.4)	1(3.7)	0	0	7(25.9)	27.00
	*χ*²	0.78	0.23	0.75	0.48	4.45	3.01	0.48	0.65	1.84	9.49
	*P*	0.68	0.91	0.82	1.00	0.06	0.13	1.00	1.00	0.41	0.91
Ethnicity	Han	51(31.5)	59(36.4)	4(2.5)	3(1.9)	3(1.9)	2(1.2)	2(1.2)	4(2.5)	34(21.0)	162
	Others	5(45.5)	4(36.4)	0	0	0	0	1(9.1)	0	1(9.1)	11
	*χ*²	0.92	0.00	0.28	0.21	0.21	0.14	3.73	0.28	0.90	6.04
	*P*	0.34	0.63	1.00	1.00	1.00	1.00	0.18	1.00	0.47	0.60
Marita Status	Unmarried	30(31.6)	41(43.2)	4(4.2)	1(1.1)	0	1(1.1)	2(2.1)	3(3.2)	13(13.7)	95
	Married or with Spouse	17(30.9)	15(27.3)	0	1(1.8)	3(5.5)	1(1.8)	0	1(1.8)	17(30.9)	55
	Divorced or Widowed	9(39.1)	7(30.4)	0	1(4.3)	0	0	1(4.3)	0	5(21.7)	23
	*χ*²	0.63	4.12	2.21	1.83	4.88	0.82	2.15	2.21	6.37	20.17
	*P*	0.74	0.14	0.36	0.38	0.08	1.00	0.24	0.36	0.04	0.10
Educational Attainment	Junior High School and Below	19(31.1)	22(36.1)	0	0	2(3.3)	0	1(1.6)	1(1.6)	16(26.2)	61
	High School or Secondary Technical School	25(34.7)	24(33.3)	4(5.6)	2(2.8)	0	1(1.4)	2(2.8)	1(1.4)	13(18.1)	72
	College and Above	12(30.0)	17(42.5)	0	1(2.5)	1(2.5)	1(2.5)	0	2(5)	6(15)	40
	*χ*²	0.33	0.96	4.09	1.78	2.45	1.63	0.93	1.69	2.13	14.80
	*P*	0.85	0.63	0.08	0.45	0.33	0.70	0.79	0.45	0.32	0.44
Occupation	Student	11(39.3)	9(32.1)	1(3.6)	1(3.6)	0	0	1(3.6)	1(3.6)	4(14.3)	28
	Worker/Farmer	19(33.9)	18(32.1)	1(1.8)	0	1(1.8)	1(1.8)	1(1.8)	2(3.6)	13(23.2)	56
	Service Industry/Clerk	10(33.3)	10(33.3)	2(6.7)	1(3.3)	0	0	0	0	7(23.3)	30
	Housework and Unemployed	6(13.3)	24(53.3)	0	0	1(2.2)	1(2.2)	1(2.2)	1(2.2)	11(24.4)	45
	Others	10(71.4)	2(14.3)	0	1(7.1)	1(7.1)	0	0	0	0	14
	*χ*²	17.84	8.97	3.54	5.44	3.25	2.04	2.95	1.60	5.24	39.17
	*P*	0.00	0.06	0.40	0.07	0.48	1.00	0.57	0.88	0.26	0.18
CD4 Count (cells/mm³)	201–349	24(30.0)	31(38.8)	2(2.5)	1(1.3)	2(2.5)	2(2.5)	0	3(3.8)	15(18.8)	80
	350–499	17(32.1)	16(30.2)	0	0	1(1.9)	0	3(5.7)	1(1.9)	15(28.3)	53
	≥ 500	15(37.5)	16(40.0)	2(5.0)	2(5.0)	0	0	0	0	5(12.5)	40
	*χ*²	0.73	1.31	2.38	2.80	0.83	1.48	4.76	1.24	3.57	17.34
	*P*	0.68	0.52	0.27	0.23	0.80	0.50	0.04	0.69	0.16	0.22
Viral Load (copies/mL)	≤ 10,000	13(25.5)	24(47.1)	0	1(2.0)	0	1(2.0)	1(2.0)	2(3.9)	9(17.6)	51
	10,000- 100,000	3(60.0)	1(20.0)	0	0	0	0	0	0	1(20.0)	5
	≥ 100,000	40(34.2)	38(32.5)	4(3.4)	2(1.7)	3(2.6)	1(0.9)	2(1.7)	2(1.7)	25(21.4)	117
	*χ*²	3.03	3.68	1.92	1.11	1.68	1.94	1.11	1.61	0.43	13.95
	*P*	0.21	0.17	0.39	1.00	0.59	0.54	1.00	0.63	0.87	0.72

### TDR analysis

The overall resistance rate was 7.51% (13/173), which was at a medium prevalence level. There were 4 cases with protease inhibitors (PIs) resistance, with a resistance rate of 2.31%; 1.16% (2/173) had nucleoside reverse transcriptase inhibitors (NRTIs) resistance; 2.31% (4/173) had non-nucleoside reverse transcriptase inhibitors (NNRTIs) resistance; and there were 5 cases with integrase strand transfer inhibitors (INSTIs) resistance, with a resistance rate of 2.89%. Notably, one case exhibited simultaneous resistance to NRTIs, NNRTIs, and INSTIs (Table 3).

**Table 3:** TDR distribution and resistance rate.

Types of Drug-resistant Mutation	Sequences with TDR	Resistance Rate
Overall Drug-resistant Mutations	13	7.51%
Mutations Related to PIs	4	2.31%
Mutations Related to NRTIs	2	1.16%
Mutations Related to NNRTIs	4	2.31%
Mutations Related to INSTIs	5	2.89%
Mutations Related to NRTIs+ NNRTIs + INSTIs	1	0.58%

Among the 25 ART drugs (8 PIs, 7 NRTIs, 5 NNRTIs, 5 INSTIs) listed in the Stanford HIV Drug Resistance Database, 23 drugs showed low to high level resistance except Darunavir (DRV) and emtricitabine (ETR). The highest resistance rate among PIs was found for Nelfinavir (NFV) (4/173, 2.31%), and 50% of them were high-level resistant. This was followed by Atazanavir (ATV) (2/173, 1.16%), Indinavir (IDV) (2/173, 1.16%), and Saquinavir (SQV) (2/173, 1.16%). Among NRTIs, the drugs with the highest resistance rate were Abacavir (ABC) (2/173, 1.16%), Stavudine (D4T) (2/173, 1.16%), Didanosine (DDI) (2/173, 1.16%), and Tenofovir (TDF) (2/173, 1.16%). Among NNRTIs, the drugs with the highest resistance rate were Efavirenz (EFV) (3/173, 1.73%) and Nevirapine (NVP) (3/173, 1.73%). The resistance rates to EFV and NVP were 66.7% (2/3) and 100% (3/3), respectively. Among INSTIs, the drug with the highest resistance rate was Elvitegravir (EVG) (5/173, 2.89%). The most common mutation sites were G163R (2/173, 1.16%) and R263RK (2/173, 1.16%). It is worth noting that the rate of resistance to INSTIs is slightly higher than other three classes of drugs Table 4.

**Table 4:** HIV-1 gene mutations and drug resistance in this study.

Drug	Number of Resistant Cases	Proportion (%)	Primary and Secondary Resistance Mutations
**PIs**	4	2.31	
ATV	2	1.16	M46I, M46MGRV, M46MV, N88NS, Q58QE, L90LM
DRV	0	0.00	
FPV	1	0.58	
IDV	2	1.16	
LPV	1	0.58	
NFV	4	2.31	
SQV	2	1.16	
TPV	1	0.58	
**NRTIs**	2	1.16	
ABC	2	1.16	
AZT	1	0.58	K65R, D67N, K70E
D4T	2	1.16	
DDI	2	1.16	
FTC	1	0.58	
3TC	1	0.58	
TDF	2	1.16	
**NNRTIs**	4	2.31	
DOR	2	1.16	
EFV	3	1.73	K103N, V106M, V106VA, E138G, V179D, V179E, G190A
ETR	0	0.00	
NVP	3	1.73	
RPV	2	1.16	
**INSTIs**	5	2.89	
BIC	3	1.73	
CAB	3	1.73	S153SF, G163R, R263RK
DTG	3	1.73	
EVG	5	2.89	
RAL	4	2.31	
Total	13	7.51	

### Distribution of resistant cases in different subtype populations

Among 12 HIV subtypes, CRF55_01B exhibited the highest drug resistance rate (25.0%). Regarding specific drug classes, CRF01_AE showed the highest resistance rates to PIs (3.6%) and NRTIs (1.8%), while CRF55_01B demonstrated the highest resistance rates to NNRTIs (25.0%). For INSTIs, the resistance rate of CRF01_AE was the highest, 7.1%. However, chi-square test results indicated that these differences in resistance rates across subtypes lacked statistical significance (*P* > 0.05).

Regional distribution of drug-resistant mutations.

Among 173 recently infected individuals from 11 cities of Hebei Province, Baoding had the highest proportion of HIV-1 strains with drug-resistant mutations (45.00%, 9/20), followed by Hengshui (41.67%, 5/12), Xingtai (38.46%, 5/13), Cangzhou (35.00%, 7/20), Handan (33.33%, 2/6), etc. Shijiazhuang (10.00%, 3/30) and Chengde (10.00%, 1/10) had the lowest proportion of such mutations. The chi-square test indicated that the regional distribution of these resistance mutations in the cities show no statistically significant difference (χ² = 14.17, *P* = 0.163). Consistent with this, the geographical distribution of the 13 TDR patients also showed no statistical significance (*P* = 0.642). These results suggest that the occurrence of HIV-1 drug resistance mutations and the distribution of TDR are random across the studied cities (Table 5).

**Table 5:** Distribution of HIV-1 drug-resistant mutations in the cities of Hebei Province.

City	Total Population	Number of Resistance Mutations	Percentage (%)	Number of TDR	Percentage (%)
Shijiazhuang	30	3	10.00	1	3.33
Tangshan	21	4	19.05	2	9.52
Baoding	20	9	45.00	3	15.00
Cangzhou	20	7	35.00	0	0.00
Zhangjiakou	18	5	27.78	2	11.11
Qinhuangdao	13	2	15.38	0	0.00
Xingtai	13	5	38.46	1	7.69
Hengshui	12	5	41.67	0	0.00
Chengde	10	1	10.00	1	10.00
Langfang	10	2	20.00	1	10.00
Handan	6	2	33.33	2	33.33
Total	173	45	26.01	13	7.51

### Molecular transmission networks

**Fig. 3:**
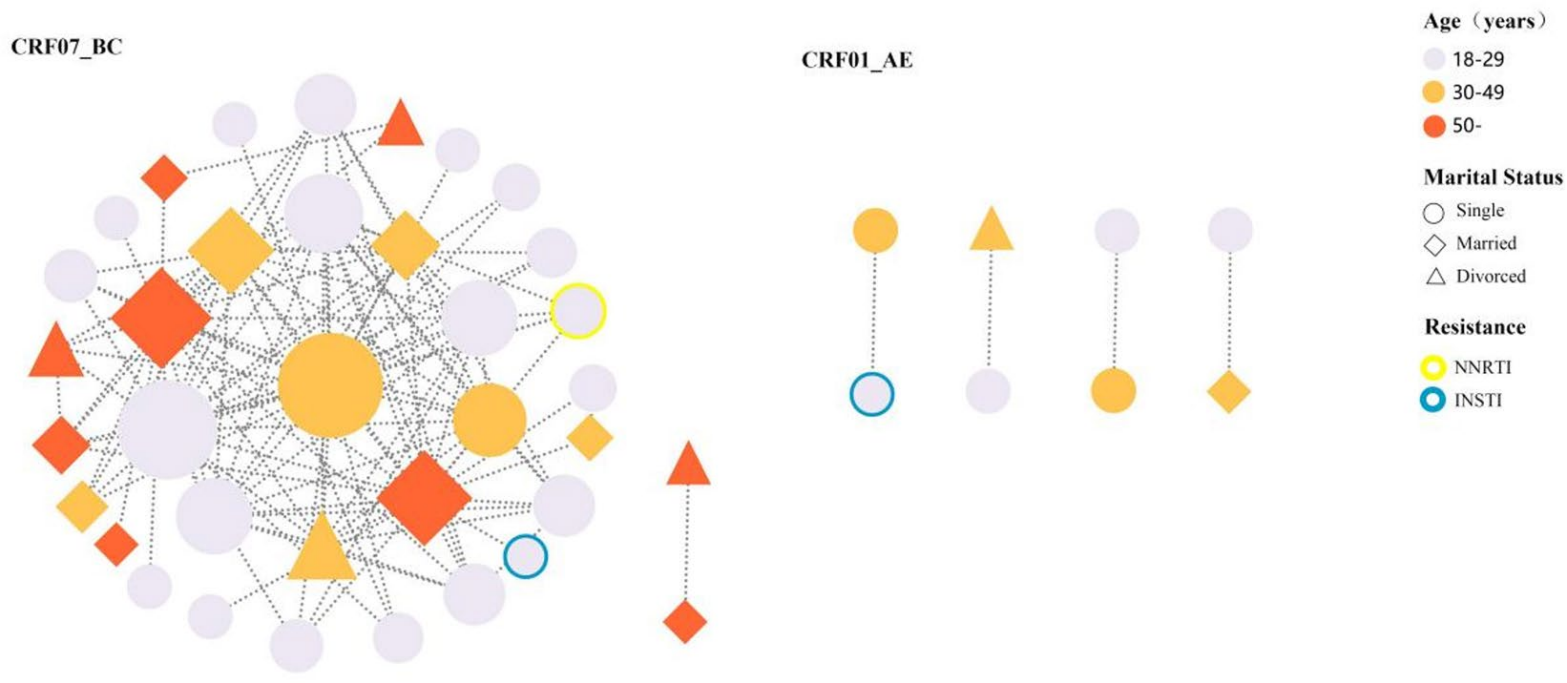
Molecular transmission of HIV-1 among recently infected individuals.

Molecular transmission networks (Fig. 3) indicated that 44 of 173 were circulating in the networks. And, the recently infected individuals with age 18–29 (45.45%, 20/44), CRF07_BC (88.%, 36/44) and unmarried (50.00%, 22/44) were included in networks. And, the CRF07_BC cluster was the largest cluster, containing 34 nodes and 119 lines. In the CRF07_BC cluster, two recently infected individuals contained two mutations (E138G and G163R), resistant to RPV and EVG/RAL, respectively.

## Discussion

A comparison with the subtype prevalence among newly diagnosed HIV-1 infections in Hebei Province between 2020–2022^9,10^ revealed that the number and types of HIV-1 subtypes prevalent in the MSM population in Hebei Province in 2023 were almost the same. But, the proportion of different subtypes was not consistent: in our reports in 2018–2022, CRF01_AE (51.9%), CRF07_BC (30.4%) and B (6.2%) were the most predominant subtypes among MSM, however CRF07_BC (36.4%) in this study has been the predominant subtype, exceeding CRF01_AE (32.3%) in 2018–2022, sharing similarities with that in China^11^. Particularly, URFs accounted for 20.2% has been the third dominant subtype in this study, significantly higher than that (5.8%) in 2018–2022. The epidemic characteristics of different subtypes among recently infected MSM in this study suggests that HIV-1 subtypes will present significant change in the future.

The most recent TDR survey on recently infected HIV-1 MSM in Hebei Province was conducted a decade ago^12^. This study added recent data on TDR prevalence by conducting drug - resistance studies on recently infected MSM. Compared with that before ten years ago, the TDR prevalence significantly among MSM increased from 2.08% in 2015 to 7.51% in 2023 in Hebei, obviously lower than those in USA (18.9%)^13^ and Germany (17.8%)^14^. The individual-class resistance rate of PIs (2.31%), NRTIs (1.16%), and NNRTIs (2.31%) was lower than those (PIs,4.2%; NRTIs ,6.9%; NNRTIs, 12.0%) in USA^13^. However, the prevalence of INSTI-TDR (2.89%) was higher than those of USA (0.8%), Beijing (0.62%)^15^, Jiangsu (1.7%)^16^, lower than higher partial HIV prevalence province such as Yunnan (5.7%)^17^ and Guangxi (3.1%)^18^. This suggests that the circulating of HIV-1 TDR strains among recently infected MSM will become the main obstacle in the fight against AIDS. Compared with our previous reports (8.3%) among newly diagnosed treatment-naïve HIV-1 individuals in 2018–2022^19^, the drug resistance prevalence was lower among recently infected individuals in 2023.

INSTIs have been recommended as agents of therapeutic regimes in many countries and regions around the world, including China, because of their high efficiency, good tolerability, and fewer drug - drug interactions^20,21^. However, since it has not yet been listed as a free antiviral treatment drug in China, its utilization rate is much lower than that of free treatment regimens such as NRTIs, NNRTIs, LPV/r and so on provided by China government^22^. Currently, the level of integrase resistance among recently infected MSM in Hebei Province is low. Thus, it can be recommended to eligible individuals, but continuous resistance monitoring is still necessary. The fact that INSTIs resistance has been quietly prevalent requires us to consistently test for resistance to the integrase region in the future.

Furthermore, molecular transmission networks identified that youthful individuals aged 18–29, accounting for 45.45%, were circulating in networks. The most of them were unmarried MSM, mainly circulating in the largest CRF07_BC cluster. This reveals that the largest CRF07_BC cluster was very active. And, the CRF07_BC strains resistant to INSTI (EVG/RAL) and NNRTI (RPV) have spread into this population. Currently, MSM is the most predominant transmission route and the prevalence of CRF07_BC is increasing in the Hebei province, suggesting that it is very important for us to take measures to control the diffusing of the resistance strains.

In China, with a treatment-seeking population of over one million, if HIV-1 drug-resistant strains become dominant, these dominant drug-resistant strains will be transmittd to general populations, creating a vicious cycle that is difficult to control. Therefore, apart from monitoring drug resistance in patients who have started treatment, detecting drug resistance in newly infected HIV-1 individuals is of great public-health significance. In the future, we should continue to monitor drug resistance among individuals recently infected with HIV-1, including the resistance related to integrase region, and stay informed about its development. This will enable us to evaluate the effectiveness of the current treatment regimen and make appropriate adjustments to second-line therapies and public-health measures.

It is crucial to emphasize that this study is strictly confined to individuals with recently acquired HIV-1 infection. In contrast, most published data from China were derived from newly reported cases, which were predominantly composed of chronic infections. This fundamental difference in case definitions precludes meaningful direct comparisons and underscores a major methodological gap in current surveillance. In the absence of a nationally representative cohort of recently infected persons, we relied on longitudinal local data to chart temporal trends. The resulting dataset provides a critical benchmark for future standardized national surveillance of recently acquired HIV-1 infections and their resistance profiles.

This study provides a preliminary description of drug-resistance characteristics among recently HIV-1-infected MSM in Hebei Province, establishing a data foundation for subsequent research. Continuous surveillance of drug resistance, including integrase inhibitor resistance, will also be maintained. Together, these efforts will allow us to evaluate the efficacy of current treatment strategies and thus inform targeted refinements to second-line therapies and broader public-health intervention.

Limitations: Subtyping results were classified as low confidence if inconsistencies between coding regions or detectable recombination within a single region were observed. Perhaps because of the small sample numbers, the chi-square test revealed no statistically significant difference in the regional distribution of these resistance mutations.

## Materials and methods

### Study population and data Sources‌

This cross-sectional study analyzed all recently infected HIV-1 MSM in Hebei Province, China, who were recorded in the 2023 surveillance database of the CACDIMS. Written informed consent has been obtained from the participants to publish this paper.

Inclusion Criteria:

(1) Participants who have not received ART.

(2) No history of pre-exposure prophylaxis (PrEP) or post-exposure prophylaxis (PEP).

(3) Current residence address is in Hebei Province.

(4) The route of transmission is “MSM”.

(5) Test of limiting antigen avidity enzyme immunoassay (LAg-Avidity EIA) is positive.

(6)Viral Load ≧ 1000 copies/mL.

(7) CD4 cell counts > 200 cells/mm³.

Exclusion Criteria:

(1) Incomplete personal information.

(2) Do not meet any of the inclusion criteria.

### HIV-1 RNA extraction, amplification and sequencing

Plasma RNA was extracted using the Nucleic Acid Extraction Kit (Cat. No. 2402007) from Zybio Inc. After reverse transcription, the protease, reverse transcriptase (1.3 kb), and integrase (0.8 kb) gene coding regions within HIV-1 *pol* were amplified using an in-house method. Subsequently, the amplified products were purified and then sequenced using the Sanger method.

### Subtype and drug resistance analysis

The successfully sequenced sequences were edited, spliced, and corrected, and then submitted to the Stanford HIV Resistance Database (https://hivdb.stanford.edu/) for HIV − 1 drug resistance and subtyping. This process enabled the identification of resistance mutation sites and the degree of resistance. The systematic scale categorizes the degree of resistance as S (susceptible), P (potential low - level drug resistance), L (low - level drug resistance), I (intermediate drug resistance), and H (high - level drug resistance). Scores of L ( ≧ 15) and above are recognized as indicating drug resistance. HIV-1 different subtypes were judged via constructing phylogenetic tree and HIV blast (https://www.hiv.lanl.gov/content/sequence/BASIC_BLAST/basic_blast.html), and suspected subtypes were further analyzed using recombination identification tools (jpHMM, RIP 3.0, and SimPlot 3.5.1) to confirm unique recombinant forms (URFs).

### Construction of transmission networks

Molecular transmission networks were constructed based on the study pol sequences. Pairwise genetic distances were calculated using HYPHY 2.2.4 with a Tamura-Nei 93 (TN93) model. We selected a genetic distance threshold of 0.015 substitutions/site to construct networks because this threshold is consistent with recent and rapid transmission. Molecular transmission networks were visualized using HIV-Trace-1.5.0.

### Data collation and statistical analysis

Data collation and statistical analysis were performed using Microsoft Excel 2021. SPSS 23.0 (IBM SPSS Statistics, USA) was used to conduct the chi - square test, with statistical significance defined as a P - value less than 0.05.

## Conclusions

This study provides the first comprehensive TDR profile for recently infected population in Hebei, offering critical insights for regional HIV-1 management.

## Data Availability

The datasets used and analyzed during the current study are available in the GenBank with the accession numbers PV874282-PV874300, PV929852-PV929871, PV916236-PV916239, PV921469-PV921483, PV921576-PV921588, PV927316-PV927333, PV927348-PV927372, PV929852-PV929871.
